# Sodium iodide symporter (NIS) in extrathyroidal malignancies: focus on breast and urological cancer

**DOI:** 10.1186/1471-2407-14-303

**Published:** 2014-04-30

**Authors:** Salvatore Micali, Stefania Bulotta, Cinzia Puppin, Angelo Territo, Michele Navarra, Giampaolo Bianchi, Giuseppe Damante, Sebastiano Filetti, Diego Russo

**Affiliations:** 1 Department of Urology, University of Modena and Reggio Emilia, Via Largo del Pozzo, 71, Modena 41100, Italy; 2 Department of Health Sciences, University of Catanzaro ‘Magna Graecia’, Catanzaro 88100, Italy; 3 Department of Medical and Biological Sciences, University of Udine, Udine 33100, Italy; 4 Department of Drug Sciences and Products for Health, University of Messina, Messina 98100, Italy; 5 Department of Internal Medicine and Medical Specialties, University of Rome ‘Sapienza’, Rome 00100, Italy

**Keywords:** Sodium iodide symporter (NIS), Extrathyroidal tissues, Breast cancer, Urological malignancies, Gene therapy

## Abstract

**Background:**

Expression and function of sodium iodide symporter (NIS) is requisite for efficient iodide transport in thyrocytes, and its presence in cancer cells allows the use of radioiodine as a diagnostic and therapeutic tool in thyroid neoplasia. Discovery of NIS expression in extrathyroidal tissues, including transformed cells, has opened a novel field of research regarding NIS-expressing extrathyroidal neoplasia. Indeed, expression of NIS may be used as a biomarker for diagnostic, prognostic, and therapeutic purposes. Moreover, stimulation of endogenous NIS expression may permit the radioiodine treatment of extrathyroidal lesions by concentrating this radioisotope.

**Results:**

This review describes recent findings in NIS research in extrathyroidal malignancies, focusing on breast and urological cancer, emphasizing the most relevant developments that may have clinical impact.

**Conclusions:**

Given the recent progress in the study of NIS regulation as molecular basis for new therapeutic approaches in extrathyroidal cancers, particular attention is given to studies regarding the relationship between NIS and clinical-pathological aspects of the tumors and the regulation of NIS expression in the experimental models.

## Introduction

**Figure 1:**
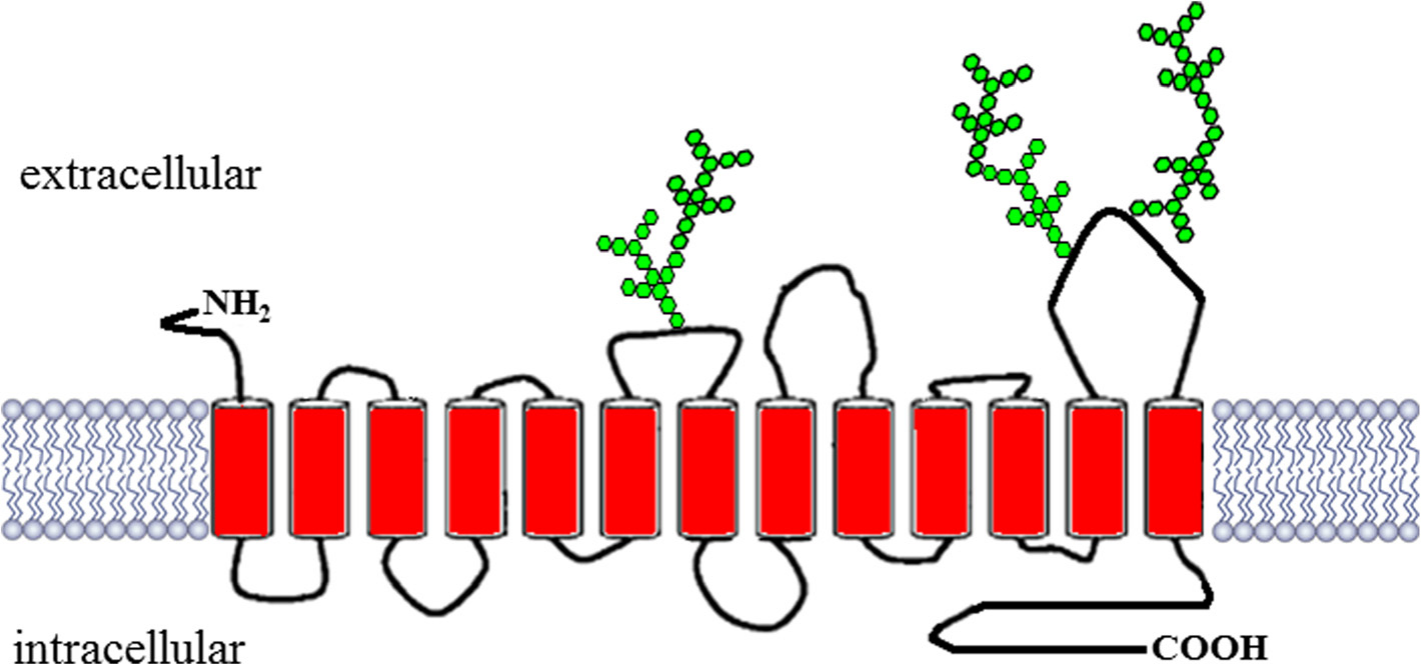
**NIS schematic model.** The transporter contains 13 transmembrane domain (in red) and 3 N-linked glycosylation sequences (in green).

The sodium iodide symporter (NIS) is a glycosylated protein with 13 trans-membrane domains, belonging to the solute carrier family [1,2] (Figure 1). It is able to transport 2 Na^+^ and one I^-^ through the membranes, depending on the Na^+^ gradient maintained by Na^+^/K^+^ ATPase [3]. The highest expression levels are detectable in the thyroid, where is located in the basolateral membrane of the thyrocytes [3]. NIS activity is necessary to provide the iodide concentration gradient inside thyroid cells, used for the synthesis of thyroid hormones in a multistep process requiring the action of pendrin, thyroid peroxidase (TPO), dual oxidase-2, and thyroglobulin. The thyroid stimulating hormone (TSH) is the main regulator of the iodide transport in the thyrocytes, and it does so by acting on NIS transcription, NIS protein half-life, and its translocation to the thyrocyte basal plasma membranes [3-5].

### NIS expression in thyroid cancer and radioiodide therapy

The presence of NIS in thyroid cancer cells, by allowing highly efficient iodide accumulation, is exploited for the use of radioactive substrates of NIS for diagnostic and therapeutic purposes. Thus, when functional NIS expression is maintained in metastatic lesions, radioiodide-131 (^131^I) administered after total thyroidectomy permits selective ablation of neoplastic tissue. However, expression of endogenous NIS and subsequent radioiodide uptake is often reduced in thyroid cancer, especially in metastatic tissue [6]. Stimulation of NIS expression is therefore required prior to ^131^I administration, and is currently obtained by elevating TSH levels [7,8]. However, there are some tumors, especially the less differentiated ones, that are unresponsive to such a treatment [7,8]. An increase of NIS expression, and subsequent iodide concentration ability, has been successfully obtained in thyroid tumor cells also by using inhibitors of some oncogenic signaling pathways in *in vivo* and *in vitro* experimental models [9]. Indeed, restoration of *NIS* expression by differentiation-inducible agents, acting by genetic or epigenetic mechanisms [9,10], or enhancement of iodide uptake by using potential NIS translocation stimulators, have been reported in less differentiated thyroid cancer cells [11]. Altogether, such findings provide a promising basis to extend the radioiodine approach for those tumors that are still nonresponsive to radioiodine treatment.

### NIS expression in extrathyroidal tissues

Various extra-thyroidal tissues express NIS at mRNA and/or protein levels [12]. By using immunohistochemistry, Wapnir and coworkers showed that several normal tissues, including bladder, colon, endometrium, kidney, prostate, and pancreas, expressed NIS protein. However, plasma membrane immunopositivity was confirmed only in salivary ductal, gastric mucosa, and lactating mammary cells [13] (Table 1). Iodide uptake was also reported in choroid plexus cells in a pre-NIS era [14-16].

**Table 1:** NIS expression in normal extrathyroidal tissues

**Tissues**	**mRNA**	Protein (*)	**References**
Lacrimal glands		**+**	[17]
Salivary glands		+	[13,18-20]
Stomach	**+**	**+**	[13,18,19]
Colon		**+**	[13]
Testis	**+**	**+**	[21]
Endometrium		**+**	[13]
Placenta	**+**	**+**	[22]
Lactating mammary		**+**	[13,23]

(*) only when detected in the plasma membrane.

NIS acts in salivary glands, stomach, and intestine to provide efficient adsorption of iodide contained in the food [3]. While salivary glands (mainly the parotid glands) and stomach cells transfer iodide from the bloodstream to the lumen of the gastrointestinal tract [24], intestines take the iodide from the lumen to transport it into circulation [25]. For this reason NIS is expressed on the basolateral membrane of salivary ductal and gastric mucosa cells [19,26], and, vice-versa, on the apical membrane of the brush border of small intestine [25].

Lactating mammary glands are able to provide a sufficient amount of iodide in the milk to reach a concentration of approximately 150 μg/L [27]. This is obtained thanks to abundant NIS expression on the basolateral membrane of the alveolar cells [28], which mediates the transfer from the bloodstream into milk. Stimulation of NIS expression occurs during lactation due to increased levels of various hormones, including oxytocin, prolactin, and estrogens [23,28]. In contrast, non-lactating normal breast tissue does not express NIS protein and is not able to accumulate iodine, unless pathological conditions like hyperprolactinoemia occur [29]. Indeed, Bruno and coworkers [12], investigating a series of patients who underwent whole-body ^131^I following the administration of high doses of ^131^I for thyroid carcinoma, demonstrated that only a very small fraction of normal breast tissues presented efficient iodine uptake.

NIS also operates in placental cells, contributing to the transfer of iodide from the mother to the fetal circulation [22].

Finally, the presence of NIS mRNA and protein was demonstrated at low levels also in fetal and adult human testicular tissues [21]. Expression of NIS in the germinal cells may represent the molecular basis for the concentration of radioiodine, responsible for the alterations observed in male patients undergoing this treatment for thyroid cancer. However, the low amount of NIS in the plasma membranes, as well as the presumable rapid efflux of radioiodide due to the absence of an organification machinery in testicular cells, may explain the presence of only transient alterations observed in these patients [21].

### NIS expression and radioiodide uptake in extrathyroidal tumors

The crucial role of radioiodide-based therapy in thyroid cancer and the characterization of the molecular basis of iodide transport following the cloning of *NIS*, including its detection in some extrathyroidal tissues, has encouraged a large series of studies aimed to try to extend radioiodine treatment even to extrathyroidal tumors after induction of NIS expression.

When this strategy is adopted for the treatment of extrathyroidal tumors, it becomes necessary to prevent radioiodide uptake and concentration in normal thyrocytes. Selective downregulation of NIS expression, as well inhibition of organification, has been successfully obtained by using combination of T3 and methimazole [30]. In addition, also high doses of iodide are able to downregulate NIS expression in normal thyrocytes.

The two strategies currently explored to induce NIS expression in cancer cells include the transfer of *NIS* gene using vectors (mainly viruses) and constructs able to ensure the selective expression in tumor cells, or, alternatively, the stimulation of the expression of a functional endogenous NIS.

In the next sections, we will describe recent findings regarding NIS expression in extrathyroidal malignancies, focusing on breast and urological cancers, and emphasizing the most relevant developments in both gene therapy and endogenous NIS stimulation strategies.

### NIS and breast cancer

#### NIS expression in breast cancer

The demonstration of NIS presence in lactating breast [23] has suggested that this protein could be expressed also in breast cancer (BC). Accordingly, in the seminal study in which NIS expression in lactating breast was discovered, it was shown that this protein is expressed in more than 80% of both invasive and *in situ* BCs [23]. However, both plasma membrane and intracellular immunohistochemical signal was detected (Table 2), which is in contrast to the only basolateral membrane signal detected in lactating breast. The notion that the NIS protein is expressed in a large number of breast carcinomas was confirmed by the same group by investigating a larger cohort of samples [13]. In this study it was found that NIS is also expressed in about 80% of fibroadenomas. Again, in breast carcinomas, the NIS protein was predominantly located in the cytoplasm, suggesting that in BC a deficiency of NIS trafficking from cytoplasm to plasma membrane occurs. High levels of NIS positivity in BC by immunostaining has also been described in other studies [31,32]. It should be pointed out, however, that such a large positivity, when obtained by immunohistochemistry in the cytoplasmic compartment, could be due to non-specific staining [33]. In order to understand the molecular basis of NIS inability to target the plasma membrane in a large fraction of BC, genes whose expression is associated to NIS plasma membrane localization have been recently identified by microarray analysis [34]. Interestingly, the cysteinyl-tRNA synthetase gene is highly associated with cell surface NIS protein levels only in the estrogen receptor (ER)-positive BC subtype, suggesting that molecular mechanisms responsible for reduced plasma membrane localization of NIS may be different in a distinct subtype of BC [34].

**Table 2:** NIS expression in breast cancer tissues

**No of specimens**	**mRNA**	**Protein positive (%)**	**References**
45	n.d.	69	[23]
50	n.d.	90	[31]
12	+	100	[35]
27	n.d.	30	[30]
23	n.d.	65	[36]
28	+	7	[34]
75	+	n.d.	[37]
32	n.d.	92	[32]

n.d.: not determined.

Triple-negative BCs (TNBCs) are defined by the absence of ER, the progesterone receptor (PR), and the human epidermal growth factor receptor 2 (HER2) expression [38]. Because of absence of ER, PR, and HER2, TNBC cannot be treated by hormonal therapy or HER2-targeting compounds, leaving chemotherapy as the only therapeutic tool. Patients with this disease have a worse outcome than patients with other BC subtypes [38,39]. It has been shown that NIS is expressed in about 65% of TNBCs and that in a fraction of them a strong plasma membrane localization is present [40]. Accordingly, in the same study, efficient iodine uptake was detected by ^123^I scintigraphy in a patient. The notion that the NIS protein expressed in BC is able to allow radioiodine uptake has been reported in other studies as well. In fact, by studying women with infiltrating duct carcinoma, high NIS expression at both transcriptional and translational level and its ability to transport iodine in cancer tissue has been demonstrated [35]. Recently, Damle and coworkers reported that the radioiodine uptake in breast cancer specimens was significantly higher as compared to that observed in the normal tissue from the same patients [41]. In this study, 50% of breast cancer samples were positive for radioiodine uptake as well as *NIS* gene expression [41].

Expression and function of NIS has been investigated also in metastatic BC. Wapnir and coworkers investigated 23 patients with metastasis predominantly at the level of lung, liver, bone, and lymph node/soft tissues [30]. Eight of these subjects showed protein NIS expression, and iodide uptake was noted in two of eight NIS-expressing tumors. The same group has more recently investigated NIS expression in brain metastasis by immunohistochemistry [40]. In 75% of cases a predominant cytoplasmic signal was detected; however, plasma membrane immunoreactivity was detected only in 23.8% of NIS-positive samples. Altogether these data would indicate that NIS protein is correctly located and is able to accumulate iodine only in a small fraction of BC metastasis.

Besides immunohistochemical studies, high expression of NIS mRNA has been shown by quantitative reverse transcriptase polymerase chain reaction (RT-PCR) evaluation. Oh and coworkers have shown that *NIS* gene expression was present in approximately one-third of BC tissues, and no relationship was found between NIS mRNA levels and hormonal receptors expression [42]. More recently, Ryan et al. confirmed that NIS expression levels are significantly higher in BC and fibroadenoma than in normal tissue, with the highest levels of NIS mRNA observed in fibroadenoma tissues [37]. At present, detection of NIS expression levels has no prognostic value: in fact no significant relationship has been detected between NIS mRNA levels and clinical characteristics of the tumors [37]. In addition, immunohistochemistry of a subset of tumor tissues in the same cohort confirmed the presence of NIS protein both in selected malignant carcinomas and benign fibroadenomas [37].

#### NIS-based gene therapy

A strategy attempted to achieve significant radioiodine uptake by the BC cells is using gene therapy to introduce an "active" exogenous *NIS* gene. Montiel-Equihua and coworkers have generated a replication-incompetent adenovirus, AdSERE, in which the expression of NIS is directed by an estrogen-responsive promoter [43]. Therefore, this vector would be active only in ER-positive BC (about 60% of all BC). *In vitro*, AdSERE mediated human NIS expression and iodide uptake in ER+ cell lines (MCF7 and ZR75-1). Moreover, the authors show that ZR75-1 AdSERE-positive xenografts in nude mice can be imaged after ^99m^Tc injection and their growth suppressed with therapeutic doses of ^131^I [43]. The use of a non-replicative adenovirus has been recently reported by the Santisteban group [44]. In this virus, NIS transcription is driven by promoters of human telomerase subunits RNA (hTR) and human telomerase reverse transcriptase (hTERT). Telomerase is a ribonucleoprotein that is essential in most human cancers but is not expressed in most normal tissues [45-47]. Thus, hTR and hTERT promoters would be active only in cancer cells. When the BC cell line MDA-MB-231 was infected by this virus, expression of NIS protein, iodine uptake, as well as reduced cell survival after radioiodine administration was observed. A conditionally replicating adenovirus (CRAd) in which the E1a gene is driven by the tumor-specific promoter Mucin 1 (MUC-1) has also been generated [48]. This virus can efficiently replicate only in MUC-1 overexpressing cells, including BC cells [49]. In addition, this virus contains the transcriptional cassette RSV promoter-h*NIS*cDNAbGH polyA in the E3 region, which permits NIS to express at high levels. After infection of the MUC-1-positive BC cell line T47D, virus replication, cytolysis, and release of infective viral particles, as well as iodide uptake, were observed [48].

The increase of the exogenous, virus-mediated expression of the *NIS* gene by pharmacological treatment has been also investigated. Treatment with retinoic acid (RA) has been shown to increase NIS expression in MCF7 cells infected by a non-replicating adenovirus in which NIS expression is controlled by the potent cytomegalovirus (CMV) promoter [50]. Indeed, the CMV promoter contains an RA-responsive element [51]. A large increase of iodine uptake has been also described in virus-infected, RA-treated MCF7 cells.

#### Induction of endogenous NIS

Though NIS expression has been demonstrated in most BCs, only in very few patients would spontaneous NIS expression allow efficient radioiodine uptake. For this reason, a large body of investigation has been undertaken to identify compounds that are able to increase NIS expression, its localization in plasma membrane, and iodine uptake. The major inductor of NIS expression in breast cancer cells is certainly RA. Several compounds of the RA family stimulate NIS expression, including all-*trans* RA, 13-*cis* RA, and AGN190168, all of which are already used for medical purposes [9]. Among them, the one used most to activate NIS expression in BC cells is all-*trans* RA. NIS expression has been induced in several BC cell lines including MCF7, T47D, and BT474 [52]. Several data indicate that RA induces NIS expression primarily by activating RARβ/RXRα heterodimer receptors. Hormone-bound receptor may act through two mechanisms. The first is binding to an element located in *cis* to the *NIS* gene [9]. It has been demonstrated that in MCF7 cells, treatment by RA induce retinoic acid receptor-alpha (RARα) binding to a retinoic acid response element located in intron 2 of the *NIS* gene [53]. It must be mentioned, however, that the NIS regulation by RARα was not confirmed in a different study performed on MCF7 cells [9]. The second mechanism is activation of the phosphoinositide 3-kinase (PI3K) pathway and the p38MAPK pathway. In MCF7 cells, Ohashi and coworkers have shown that either treating cells with the PI3K inhibitor LY294002 or inducing knockdown of p85alpha (a regulatory subunit of PI3K) decreases RA-induced NIS expression. Moreover, the AKT inhibitor VIII decreases iodine uptake in MCF7 cells in a dose-dependent manner [54]. Kogai and coworkers, by using both gain and loss of function experiments, have shown that p38β plays a role in the RA-induced NIS expression increase in MCF7 cells [55]. Interestingly, in the same study it was shown that in FRTL5 thyroid cells not the β but the p38α isoform has a role in NIS control of expression. Moreover, NIS induction was also observed in mouse MCF7 xenograft [56,57], although this finding was not confirmed by another group [58]. These different results using MCF7 cells might be due to heterogeneity of this cell line [59].

In addition to gene expression, the PI3K pathway regulates NIS localization. Glycosylation of NIS protein is necessary to plasma membrane localization [60]. In MCF7 cells, overexpression of PI3K increases the non-glycosylated NIS protein [61]. In the same study, it was shown that the presence of NIS in the plasma membrane as well as iodine uptake was reduced by an active mutant of PI3K. It appears, therefore, that activation of the PI3K signaling pathways exerts opposite effects on NIS: expression is activated while NIS localization in the plasma membrane is inhibited.

Several compounds cooperate with RA in inducing NIS expression in BC cell lines. RA-induced enhancement of NIS is increased by hydrocortisone, dexamethasone, troglitazone (a peroxisome proliferator–activated receptor γ, PPARγ, agonist), histone deacetylase (HDAC) inhibitors (tricostatin A and sodium butyrate), and carbamazepine [58,62-64]. Hydrocortisone, dexamethasone, troglitazone, and carbamazepine cooperate with RA also in inducing iodine uptake. Interestingly, by using MCF7 xenografts in nude mice, it has been shown that RA alone is not able to increase iodine uptake; however, significant increase in ^123^I accumulation occurs when RA is used in combination with dexamethasone [65]. Other stimulators, such as prolactin, insulin, and insulin growth factor (IGF)-I and II, are able to increase NIS mRNA levels in MCF7 cells also in the absence of RA [66]. Fortunati and coworkers reported that the HDAC inhibitor LBH589 significantly induced NIS mRNA and protein levels as well as iodine uptake in several BC cell lines [67]. Table 3 summarizes the data regarding the stimulation of iodide uptake in breast cancer cells.

**Table 3:** Stimulators of iodide uptake in breast cancer cell lines

**Cell line**	**Stimulator**	**Mechanism of action**	**I**^**- **^**uptake (fold of induction)**	**References**
MCF7	tRA,9-*cis* RA	RAR/RXR agonist	10 ~ 13	[68]
MCF7	AGN190168	RARβ/γ agonist	10 ~ 13	[52]
MCF7	Am80	RARα/β agonist	~7	[56]
MCF7	Theophylline	PDE antagonist/P2R inhibitor	~4.7	[69]
MCF7	LBH589	HDAC inhibitor	~2.3	[67]
T47D	LBH589	HDAC inhibitor	~4.8	[67]
MDA-MB231	LBH589	HDAC inhibitor	~2.7	[67]
MCF7	Insulin	Insulin receptor	~12	[66]
MCF7	IGF-I	IGF-I receptor	~7.8	[66]
MCF7	IGF-II	IGF-II receptor	~10.3	[66]
MCF7	Prolactin	Cytosolic PKs activation	~9	[66]
MCF7	Forskolin	Adenilyl-cyclase/PKA activation	~3.1	[66]
MCF7	TPA	PKC activation	~2.6	[66]
MCF7	(Bu)2-cAMP	PKA activation	~3.4	[66]

*Abbreviations*: *tRA* trans retinoic acid, *RAR* retinoic acid receptor, *PDE* phosphodiesterase, *HDAC* histone deacetylase, *IGF* insulin growth factor, *PK* protein kinase.

### NIS and urological malignancies

#### NIS expression in prostate cancer

In 2010 Navarra et al. analyzed the expression of NIS in tissue specimens from a large series of patients with prostate adenocarcinoma [70]. They demonstrate that approximately half of prostate cancers express the NIS at both mRNA and protein levels (Table 4). In addition, NIS expression correlates with aggressive features of the tumors such as Gleason score and pathologic stage, thus suggesting the hypothesis that these changes are a result of the dedifferentiation process occurring during a late stage of malignant transformation. A quantitative evaluation of NIS protein levels, using more sensitive methods than immunohistochemistry, will provide more details on the role of NIS as biomarker for prostate cancer aggressiveness, as reported for beta-catenin using fluorescence microscopy [71]. In prostate tumor cells expressing NIS, it appears primarily in the cytosolic fraction of the acini as a result of an incomplete maturation or too low levels of expression, as hypothesized in some thyroid and breast cancers [72,73]. In any case, the observed strong staining of the cytoplasm makes it difficult to discern plasma membrane immunoreactivity, so that the presence of a functional NIS in the tumor cell plasma membranes could not be proved.

**Table 4:** NIS expression in extrathyroidal cancer tissues

**Primary cancer**	**No of specimens**	**NIS mRNA**	**NIS protein positive (%)**	**References**
Bladder	24	n.d.	42	[13]
Cervix	11	n.d.	100	[13]
Colon	75	n.d.	63	[13]
Esophagus	15	n.d.	47	[13]
	20	n.d.	20	[13]
Liver	26	+	8	[74]
	20*	+	100	[74]
Lung	58	n.d.	66	[13]
Ovary	37	n.d.	73	[13]
Pancreas	11	n.d.	64	[13]
Prostate	34	n.d.	74	[13]
Skin squamous	18	n.d.	56	[13]
Stomach	27	n.d.	59	[13]
	4	+	n.d.	[12]
Submandibular gland	3	+	n.d.	[12]
Testis	11	n.d.	9	[13]
	107	+	64	[75]
Uterus endometrium	25	n.d.	56	[13]
**Metastatic cancer**				
Liver**	15	n.d.	80	[76]
Brain***	28	n.d.	84	[40]

n.d.: not determined; *cholangiocarcinoma; **metastasis from breast, pancreas, colorectal and biliary cancers; ***metastasis from breast cancer.

A recent report, proposing the function of cytoplasmic NIS as an element of a pathway involved in tumor cell invasive capacity [76], suggests a role of cytoplasmic NIS in tumor aggressiveness, strengthening the hypothesis of using NIS expression as biomarker for defining individuals with biologically active prostate cancer.

#### NIS expression in testicular cancer

In 2003 Wapnir et al., by analyzing a few specimens of testicular tumors by immunohistochemistry, first evidenced the expression of NIS in some cores of these tumors [13]. In a larger study including a series of 107 testicular tumors, we have recently demonstrated that NIS is expressed in the plasma membrane of the large majority of seminomas and embryonal carcinomas of human testis, while it is absent in Leydig cell cancers [75]. Our data also demonstrated a significant association of the expression of NIS protein with lymphovascular invasion, a well-known marker of aggressiveness. We believe that the association between NIS expression in the tumor cells and lymphovascular invasion may reflect the different biological aggressiveness of testis tumors, suggesting the presence of NIS as an unfavorable prognostic factor. Also, its presence in the plasma membrane compartment of the tumor cells suggests that it may serve as potential carrier of radioiodine for an ablative treatment of cancer tissue.

#### NIS-based gene therapy

A successful prostate cancer xenograft model has been first described that accumulates 25–30% ID/g in the tumors [77]. For comparison, poorly differentiated thyroid cancer xenografts accumulated only 4.9-9.3% ID/g and were not effectively treatable with radioiodine [78]. A *NIS* gene delivered with an adenovirus vector and a tissue-specific gene promoter, the prostate-specific antigen gene (PSA) promoter, conferred efficient functional NIS expression in prostate cancer xenografts [79,80]. In a recent report, Trujillo and coworkers, by using a prostate tumor–specific CRAd in a xenograft model of prostate cancer, demonstrated that the efficacy of radioiodide therapy depends mainly on an efficient viral tumor spread and a decrease in the rate of the efflux of radioisotope [81]. To achieve synergistic or additive cytotoxic effects, combined treatments with *NIS* gene therapy and a tumor targeting strategy, such as utilization of an oncolytic vector [82], are also under experimentation.

#### Induction of endogenous NIS

Induction of NIS expression has been obtained *in vitro* in two testicular cancer cell lines. Findings from our laboratories revealed that NIS expression may be enhanced in vitro in a human embryonal testicular carcinoma cell line by the histone deacetylase inhibitor (HDACi) [75]. Histone acetylation is a known epigenetic mechanism of regulation of gene expression, and its alteration has been reported in many human cancers [83]. In many cell lines of thyroid and non-thyroid cancer, HDACi have been successfully tested to induce radioiodine uptake due to increased NIS expression [84-86]. The same result was obtained in the NTERA cells in our study, showing that, at least *in vitro*, embryonal testicular tumor cell susceptibility to radioiodine administration may occur, and suggesting the possibility of using radioiodine after pharmacological induction of NIS expression even in this rare tumor histotype. It is noteworthy that these drugs are being tested in clinical trials at doses compatible with those effective *in vitro*.

Recently, Maggisano et al. analyzed the effects of the HDACi suberoylanilide hydroxamic acid (SAHA) and valproic acid (VPA) on NIS expression and function in rat Leydig testicular carcinoma cells (LC540) [87]. LC540 cells were exposed to SAHA 3 μM and VPA 3 mM (alone and in combination), and NIS mRNA and protein levels were evaluated by using, respectively, real-time RT-PCR and western blotting. Also, NIS function was analyzed by iodide uptake assay. They found that both HDACi, used alone, were able to stimulate the transcription of *NIS* gene, but not its protein expression, while the association of SAHA and VPA increased both NIS transcript and protein levels, resulting in a significant enhancement of radioiodine uptake capacity of LC540 cells. These data demonstrate the presence of an epigenetic control of NIS expression in Leydig tumor cells, suggesting the possibility of using the combination of these two HDACi for a radioiodine-based treatment of these malignancies.

Considering altogether data obtained in breast, prostate and testicular cancer, an important difference seems to emerge. In fact, in testicular and prostate cancer NIS expression, evaluated by immunohistochemistry, appears to be related to the degree of dedifferentiation and aggressiveness [70,75,76]. However, such a relationship is not present in breast cancer [37]. Such a difference seems not due to different methodologies in detecting NIS expression. In fact, the lack of correlation between NIS expression and dedifferentiation was detected in breast tumors both by using RT-PCR and immunohistochemistry [37]. Thus, discrepancy observed between testicular/prostate tumors and breast tumors would depend on the difference of originating tissues. It would be relevant to test such a possibility in a single study, in which testicular, prostate and breast cancer are investigated by the same methodology.

### NIS and other malignancies

Tumors arising in different non-thyroidal organs, such as esophagus, colon, liver, pancreas, lung, ovary, and skin, showed NIS expression (Table 4), though the transporter was mainly and weakly detected only in the cytoplasm of neoplastic cells. Thus, the possibility of using radioiodide treatment in these tumors is strictly dependent on the possibility of achieving an adequate amount of NIS expression in the plasma membrane of tumor cells through stimulation of endogenous or exogenous NIS. According to the recent study of Lacoste et al. [76] (see above), the attribution to the intracellular NIS fraction of a role in tumor cell locomotion may have important implications for those tumors expressing NIS in the cytoplasmic compartment, allowing use as a biomarker of aggressiveness. However, this hypothesis is essentially based on results from only one experimental study and needs to be confirmed by other studies.

Independent of the detection or not in human tumor tissues, a *NIS* gene therapy approach has been tested in *in vitro* and *in vivo* experimental models of many types of neoplasia. As reported in Table 5, various vectors and many different tumor-specific promoters have been used to drive the tissue-specific expression of the *NIS* gene. Several replication-defective adenoviruses and negative-sense single-stranded RNA viruses that avoid their integration into the host genomes have been utilized, and specific promoters, as the hepatocarcinoma-intestine-pancreas gene (HIP), the human telomerase reverse transcriptase (hTERT) and the alpha-fetoprotein (AFP) promoters, have shown the capacity to promote NIS expression and iodide uptake in infected cancer cells of various origins [9]. The promising results obtained in such experimental models may open the way to making targeted radiotherapy feasible for these types of extrathyroidal cancers.

**Table 5:** NIS gene therapy in extrathyroidal neoplasia

**Neoplasia**	**Vector**	Combined treatments (*)	Promoter (**)	**References**
Neuroblastoma	Plasmid-polyplex		CMV	[88]
Medulloblastoma	MV	**+**		[89]
Glioma	Ad		CMV	[90]
	Retrovirus		LTR	[91]
Multiple myeloma	MV	**+**		[92]
	VSV	**+**		[93]
Melanoma	Ad		TR/TERT	[44]
Mesothelioma	MV	**+**		[94]
Colon cancer	Ad		CMV	[95]
	Ad		CMV/CEA	[96]
	Lentivirus		UbC	[97]
	Lentivirus	**+**	UbC	[98]
	Ad	+	CMV	[99]
	Ad		TERT, TR	[44]
	MV	**+**		[100]
Colorectal cancer	Ad	**+**	Wnt-responsiveTCF4	[101]
	Ad	**+**	TR	[102]
Hepatoma	Ad		HIP	[103]
	Plasmid		AFP	[104]
	Retrovirus	**+**	CMV	[105]
	Retrovirus	**+**	TERT	[106]
	Plasmid-polyplex		CMV	[107]
	NIS-MSC		CMV in MSC	[108]
	PAMAM-Ad		CMV	[109]
Pancreatic cancer	Ad		MUC1	[110]
	MV	**+**		[111]
	Ad	**+**	E3	[82]
	Ad		Survivin	[112]
Cervical cancer	Retrovirus	**+**	CMV	[105]

(*): to enhance the tumor growth inhibition; (**): tumor cells-specific promoter.

*Abbreviations*: *polyplex* synthetic polymeric vector, *CMV* cytomegalovirus, *MV* measles virus, *Ad* adenovirus, *LTR* long terminal repeat, *VSV* vesicular stomatitis virus, *TR* telomerase RNA, *TERT* telomerase reverse transcriptase, *CEA* carcinoembryonic antigen, *UbC* ubiquitin C, *Wnt* Wingless-related integration site, *TCF4* transcription factor 4, *PAMAM-Ad* adenoviral vectors after coating with synthetic poly(amidoamine) dendrimers, *HIP* hepatocarcinoma-intestine-pancreas gene, *AFP* alpha-fetoprotein, *MSC* mesenchymal stem cells, *MUC1* mucin1 gene, *E3* E3 antigen.

Currently, at variance with breast and testis cancer cells, there are only few data of stimulation of endogenous NIS expression in other tumor cells. Very recently, Guerrieri and collaborators [113] reported that in liver cancer cells the *NIS* gene is a direct target of the p53 family, suggesting that its modulation can be exploited to obtain NIS upregulation *in vivo*.

### Biological and technical limitations of NIS-based therapy in extrathyroidal tumors

For the efficacy of the NIS-based treatment of extrathyroidal malignancies, some fundamental concepts, which still represent major limitations, need to be taken into consideration: the efficacy of the treatment is strictly dependent on the biological half-life of the radioiodide in the body and its retention in the target tumors. Indeed, about 20% of the injected radioiodide dose must be concentrated for a sufficient time to obtain a complete destruction of the tumor mass [7]. Moreover, while in *normal* thyrocytes a prolonged iodide retention is assured by incorporation of the trapped iodide into thyroglobulin, in most thyroid cancer this process is less effective, resulting in a higher amount of discharge from tumor tissue [7]. Moreover, except for few tissues provided with a peroxidase activity (i.e. lactoperoxidase in mammary gland), non-thyroid cells do not possess the iodination machinery in their transcriptome. Thus, a prerequisite for the possibility of success of a NIS-based strategy in extrathyroidal tumors is to obtain an adequate amount of NIS protein expression in tumor cell plasma membranes. On this issue, lessons from research in thyroid cancer are highly informative: after a long period of discouraging results of this approach in the clinical trials, the success of radioiodine treatment by NIS-recovered expression obtained by using a novel protein-kinase inhibitor has been recently described [114] (see next section).

## Review and Conclusions

Radioiodine administration after TSH stimulation of iodide uptake is a validated treatment effective in most differentiated thyroid cancer. Its success may be likely attributed to the TSH-induced increase of NIS expression and function in the plasma membrane of thyroid cancer cells. Moreover, novel therapeutic approaches targeting the molecular pathways responsible for the loss of differentiation (and subsequent reduction of NIS) are showing promising results in those radioiodide-refractory cancers [115].

These finding justify the efforts to set up a similar strategy, radioiodine-based treatment after stimulation of NIS expression, as a reasonable approach for those extrathyroidal tumors in which NIS can be induced in the membrane of neoplastic cells. Such a therapy would present the advantage of short duration of treatment, reducing the frequency and severity of the eventual side effects.

Introduction of *exogenous NIS* into non-thyroidal cancer have demonstrated efficient tumor shrinkage by ^131^I in several *in vivo* studies [116]. The major improvement of *NIS* gene-based therapy strategy has come from the use of vectors of oncolytic viruses or replication-defective adenoviruses, thus preventing unfavorable genomic integration. Even non-viral vectors have been efficiently tested for the same purpose (see Table 5).

Also, the use of promoter-specific driving of NIS in the target tissue has been adopted in xenograft models of many tumors. After the successful test of the first construct containing the PSA promoter used to confer efficient functional NIS expression in prostate cancer xenografts [77], several other tumor-specific promoters have shown the capacity to drive NIS expression in specific tumor tissue and determine the radioiodide inhibition of tumor growth in animal experimental models. Finally, synergistic and/or additive cytotoxic effects have been achieved combining treatments with *NIS* gene therapy and other tumor targeting strategies [9]. An unresolved question is the real feasibility of the application of such a strategy on human patients.

In addition, *endogenous NIS* stimulation also appears to be a promising approach. It may take advantage of the enormous progress obtained in thyroid cancer field of research in the elucidation of the molecular mechanism that controls thyrocyte differentiation and, in particular, NIS expression. For example, inhibitors of signal transduction pathways, as PI3K/AKT inhibitors and MEK/ERK inhibitors, or HDAC inhibitors, have demonstrated the ability to enhance the functional NIS expression in some thyroid cancer, as well as non-thyroid cancer cells [85,117,118] and, very recently, a clinical pilot study has actually demonstrated the effectiveness of the MEK inhibitor selumetinib to increase radioiodide uptake in a number of patients with advanced thyroid cancer [114].

Isoform-specific signal transduction pathways are probably involved in the tissue-specific regulation of NIS expression. Thus, elucidation of the molecular mechanism underlying such regulatory pathways may contribute to achieving a further enhancement of functional NIS expression in extrathyroidal cancer tissues, expanding the application of radioiodide therapy to all NIS-expressing neoplasia.

## Abbreviations

AFP: Alpha-fetoprotein; BC: Breast cancer; CMV: Cytomegalovirus; CRAd: Conditionally replicating adenovirus; ER: Estrogen receptor; HDAC: Histone deacetylase; HDACi: Histone deacetylase inhibitor; HER2: Human epidermal growth factor receptor 2; HIP: Hepatocarcinoma-intestine-pancreas gene; hTERT: Human telomerase reverse transcriptase; hTR: Human telomerase subunits RNA; IGF: Insulin growth factor; ^131^I: Radioiodide-131; MUC-1: Mucin 1; NIS: Sodium iodide symporter; PI3K: Phosphoinositide 3-kinase; PPARγ: Peroxisome proliferator-activated receptor γ; PR: Progesterone receptor; PSA: Prostate-specific antigen gene; RA: Retinoic acid; RARα: Retinoic acid receptor-alpha; RT-PCR: Reverse transcriptase polymerase chain reaction; SAHA: Suberoylanilide hydroxamic; TNBC: Triple-negative BCs; TPO: Thyroid peroxidase; TSH: Thyroid stimulating hormone; VPA: Valproic acid.

## Competing interests

The authors declare that they have no competing interests.

## Authors’ contributions

SM and DR contributed to the conception of the idea, drafted the manuscript and critically reviewed the final manuscript; DR and SB elaborated the sections 3, 4, 7, the figure and the tables and editing the manuscript; CP, GD and MN elaborated the section 5; AT and GB elaborated the section 6; SF elaborated the sections 1 and 2 and conclusions. All authors read and approved the final manuscript.

## Pre-publication history

The pre-publication history for this paper can be accessed here:

http://www.biomedcentral.com/1471-2407/14/303/prepub
